# A Year Through the COVID-19 Pandemic: Deleterious Impact of Hormonal Contraception on Psychological Distress in Women

**DOI:** 10.3389/fpsyt.2022.835857

**Published:** 2022-03-16

**Authors:** Alexandra Brouillard, Lisa Marie Davignon, Justine Fortin, Marie France Marin

**Affiliations:** ^1^Research Center of the Institut Universitaire en Santé Mentale de Montréal, Montreal, QC, Canada; ^2^Department of Psychology, University of Quebec in Montreal, Montreal, QC, Canada

**Keywords:** COVID-19, distress, sex differences, sex hormones, hormonal contraceptives

## Abstract

**Background:**

Women are more at risk than men of suffering from psychological distress during disease outbreaks. Interestingly, no biological factors have been studied to explain this disparity in such contexts. Sex hormone variations induced by hormonal contraceptives (HC) have been associated with mental health vulnerabilities. However, most studies have examined current effects of HC without considering whether a chronic modulation of sex hormone levels could induce long-lasting effects that persist after HC cessation.

**Objectives:**

To date, the role of HC on psychological health in women during a disease outbreak is still unknown. We aimed to investigate both current and long-term effects of HC on psychological distress throughout the COVID-19 pandemic.

**Method:**

At four time points during the COVID-19 pandemic (June 2020, September 2020, December 2020, March 2021), we collected self-reported data on psychological distress, assessing symptoms of post-traumatic stress [*via* the Impact of Event Scale-Revised (IES-R)], symptoms of depression, anxiety, and stress [*via* the Depression Anxiety Stress Scales (DASS-21)]. Linear mixed models were first used to compare men (*n* = 49), naturally cycling women (*n* = 73), and women using HC (*n* = 32) across time. To examine long-lasting effects of HC, exploratory analyses were restricted to women, comparing current HC users (*n* = 32), past users (*n* = 56), and never users (*n* = 17).

**Results:**

The first model revealed that women taking HC reported stable post-traumatic stress symptoms across time, compared to naturally cycling women and men who showed a significant decrease from T1 to T2. HC users also reported greater DASS-21 total scores over time. Moreover, HC users reported higher stress and anxiety symptoms than men. In the second model, results showed that past HC users had similar anxiety levels as current HC users. These two groups reported significantly more anxiety symptoms than never users.

**Conclusion:**

HC users report increased distress during the pandemic relative to naturally cycling women and men. Our results also suggest a long-lasting effect of HC intake, highlighting the importance of considering both the current use of HC and its history. This could provide some insight into potential avenues for explaining why some women are prone to higher psychological distress than men.

## Introduction

### Mental Health Consequences of the COVID-19 Pandemic

The mental health impacts of the COVID-19 pandemic are being increasingly documented around the world (1–3). Factors related to the virus itself, such as fear of being infected or infecting others and witnessing the increasing number of victims, were reported to be associated with poorer mental health outcomes (1, 4). In addition, factors related to the disruption of basic human needs including restrictive measures such as physical and social distancing, cessation of daily activities, and working from home were also shown to impact the wellbeing of the population (2, 5). With this, literature on disease outbreaks suggests that there are strong predictors of mental health outcomes during these stressful situations, such as psychological distress (6, 7). Psychological distress is a broad construct that encompasses a range of negative psychological symptoms [e.g., anxiety, depressive, post-traumatic stress symptoms (PTSS)]. Distress may play a role in the development of psychiatric disorders, as well as in its severity (8, 9). Indeed, there is evidence that acute anxiety, depressive, and PTSS were worsened in the general population during the pandemic (10). According to a longitudinal study, this increase in mental health burden was found in individuals without pre-existing mental health diagnoses (11). In general, among various sociodemographic factors, gender was an important variable to account for individual differences with regard to the COVID-related mental health crisis (12).

### Gender Differences in Mental Health Outcomes

In response to the COVID-19 pandemic, men and women differed significantly in adapting to the new living/working conditions, which led to higher psychological stress in women compared to men (13). Moreover, women appear to have higher levels of anxiety, depressive, and post-traumatic stress symptoms than men when facing various stressors of the pandemic (14, 15). A recent longitudinal study showed that the highest levels of depression and anxiety occurred at the onset of confinement, and that being a woman was a risk factor for higher levels of these psychological distress symptoms (16). Twenty weeks later, depression and anxiety symptoms improved, perhaps because individuals adapted to the circumstances, but gender inequalities were still present as women seemed to improve faster than men (16). However, more studies are needed to understand the different trajectories of long-term psychological distress symptoms and their relationship with gender.

Reviews on expected roles in women proposed some hypotheses to explain why women are more likely to develop higher levels of psychological distress during COVID-19 (10, 17, 18). Carli (17) noted that more women lost their job, thus, putting more economic stress on them compared to men. As a matter of fact, unemployment rates were higher for women than men in several countries (e.g., United States, United Kingdom, Canada) during the pandemic (19). In addition, women are more often the ones who hold jobs categorized as essential (e.g., nursing) (20). Consequently, they were on the front lines during the pandemic, leading to more exposure to the virus, in addition to physical and psychological consequences (17). Moreover, traditionally, women are mainly the ones who take care of responsibilities at home such as cooking, cleaning, and childcare (21). The COVID-19 regulations led to the closure of many important services (e.g., educational facilities), which served to uphold this traditional view as it put high expectations on women's shoulders to take care of children and domestic tasks (17). Currently, differences between men and women regarding psychological distress outcomes during the pandemic are often attributed to gender inequalities pertaining to environmental or social factors. However, factors related to biological sex could also play a role in this disparity and have yet not been investigated (13).

### Hormonal Contraceptives and Mental Health Outcomes

Among biological mechanisms accounting for sex disparity in mental health outcomes, sex hormones variations induced by hormonal contraceptives (HC) have been targeted as a potential vulnerability factor for women (22–24). HC contain synthetic progesterone (progestin) and, in many cases, synthetic estrogen (ethinyl estradiol), which are effective for both birth control and menstrual cycle regulation (25). HC are long-acting and reversible methods that can be taken orally, by injection, under or on the skin, or in the vagina or uterus. At the contraceptive level, hormonal effects of HC lead to the inhibition of ovulation, sperm penetration, and to desynchronization of the endometrial changes necessary for implantation (25). Moreover, this contraceptive method also has effects on the brain and is thought to impact mental health (26). It has previously been shown that the hormone-induced changes modulate the hypothalamic-pituitary-adrenal (HPA) stress axis and limbic brain regions (27). Also, through their impact on key brain regions involved in emotion and its regulation, HC have been associated with fear regulation deficits (28, 29), lowered brain serotonin binding markers (30), as well as the onset of affective disorders (31, 32).

As long-acting methods, HC are frequently taken beginning in adolescence, which is a crucial period for brain development. Therefore, HC use could potentially lead to important hormonal changes that can have neural and psychological effects in the long-term (33). A study in rats showed that early use of ethinyl estradiol during development generated more anxious behaviors compared to a control group of mature male rats (34). In humans, a study showed that women who took HC during adolescence were at a greater risk of developing depression years after first HC exposure compared to women who had first used HC in adulthood (35). Among the few studies that have looked at the long-term effects of HC on the brains of women, it has been shown that there are cognitive effects that may persist for several years after cessation of hormonal use. Indeed, HC users had better performances in domains of visuospatial abilities, speed, and flexibility when compared to the group of never users, with a duration-dependent trend (36). In women who previously used HC, the duration of contraceptive use correlated positively with hippocampal and basal ganglia volumes, even though they had been off HC for 3 years on average (37). Although these studies are correlational and the effects and mechanisms have yet to be clarified, the current literature suggests that hormone alterations through HC use could have durable effects on the brains of women, and thus may potentially impact psychological health in a long-lasting manner.

To date, no COVID-19 studies have investigated the impact of HC on psychological distress outcomes. Moreover, the vast majority of existing HC studies have only considered the current effects of HC without considering whether a chronic modulation of sex hormones levels could induce long-lasting effects that would persist after HC cessation. These studies have generally compared HC users to naturally cycling (NC) women and men, without acknowledging previous intake of HC in NC women. Thus, NC grouping is solely based on current hormonal status, which implies that the potential influence of HC use in former users has not yet been explored. The present study aimed to investigate the effects of both current and previous use of HC on psychological distress throughout the COVID-19 pandemic. We hypothesized that women taking HC will exhibit greater psychological distress than NC women and men during the COVID-19 pandemic. Moreover, compared to women who never used HC, we hypothesized that women who previously used HC will show similar levels of distress than women currently using HC.

## Materials and Methods

### Participants

This project fell within the framework of a broader longitudinal study assessing various psychological and physiological reactions to the COVID-19 pandemic. Men and women recruited for this study had all previously participated in other experiments in our laboratory between 2017 and 2019. Of the 246 individuals recontacted in May 2020, 156 (63.41%) agreed to take part in this longitudinal follow-up. Two of these individuals, both women having used HC in the past, were excluded from the analyses as they were pregnant as of May 2020 (given the important hormonal changes induced by pregnancy). All things considered, our final sample was composed of 154 participants aged between 19 and 55 years old (*M* = 34.56, *SD* = 10.03). Participants were distributed as follows: 32 HC users, 73 NC women (17 never users, 56 past users), and 49 men. A HC was considered to be any contraceptive method altering an individual's hormonal status. Thus, of the 32 participants in the HC user group, 20 used a combined oral contraceptive (COC), seven used a hormonal intra-uterine device (IUD), three used a progesterone-only oral contraceptive, one used the vaginal ring, and one used the patch. Of note, participants using a non-hormonal IUD (i.e., copper) were classified as NC women. Therefore, our HC sample was mainly composed of women using a COC (62.5%). As this project was longitudinal, three participants (two HC users, one past user) changed their hormonal profile during the study (e.g., stopped using HC). Therefore, their data were excluded from our analyses from the moment they declared this change. Given that all questionnaires were completed online and that we wanted to optimize the validity of our results, it was important to ensure that participants were attentive when reading the various questionnaire items. As such, three random questions were added to the battery of questionnaires administered at each time point to verify whether participants were paying attention (e.g., were prompted by the following “*select the choice ‘Strongly agree”'*). One NC woman (past user) answered these three questions incorrectly (did not follow the prompt) at the second time point. Therefore, the participant's data for this time point were excluded. Table 1 shows the final participant distribution across the four time points of the present study.

**Table 1:** Distribution of participants at each time point.

	**NC women**		**HC users**	**Men**
	**Past users**	**Never users**		
T0	56	17	32	49
T1	55	17	32	47
T2	53	16	31/29^*^	46
T3	52	15	31	43
T4	45	12	28	37

* *31 for the IES-R and 29 for the DASS-21*.

When initially recruited between 2017 and 2019, participants had to be French-speaking (as the questionnaires administered were all in French) and free of any physical or mental health conditions. Since then, some participants developed health conditions, depression being the most prevalent. However, these participants were statistically well distributed across our different groups (see Table 2) and results did not change when re-running the analyses without these individuals. Therefore, we decided not to control for this variable in our analyses.

**Table 2:** Sample characteristics for men, women using HC, and NC women (past and never users).

**Variable**	**Men**	**HC users**	**NC women**			***P*** **-value**	
				**Past users**	**Never users**	**Model 1**	**Model 2**
Age	38.51 (9.90)	30.47 (8.91)	33.71 (9.76)	35.29 (9.02)	28.53 (10.59)	**<0.001**	**0.010**
Ethnicity—Caucasian	37 (75.51%)	32 (100%)	53 (72.60%)	47 (83.93%)	6 (35.29%)	**0.084**	**<0.001**
Education level—Bachelor's	17 (34.69%)	18 (56.25%)	34 (46.58%)	24 (42.86%)	10 (58.82%)	0.729	0.336
Income−100,000$ +	19 (38.78%)	6 (18.75%)	17 (23.29%)	13 (23.21%)	6 (35.29%)	0.413	0.209
Mental health diagnosis	5 (10.20%)	7 (21.88%)	15 (20.55%)	13 (23.21%)	2 (11.76%)	0.260	0.590
Physical health diagnosis	7 (14.29%)	6 (18.75%)	19 (26.03%)	16 (28.57%)	3 (17.65%)	0.279	0.471
Medication use	20 (40.82%)	18 (56.25%)	37 (50.68%)	31 (55.36%)	6 (35.29%)	0.356	0.304
Having children	33 (67.35%)	12 (37.50%)	41 (56.16%)	36 (64.29%)	5 (29.41%)	**0.030**	**0.009**
Full time cohabitation with children	24 (72.73%)	11 (91.67%)	36 (90%)	31 (88.57%)	5 (100%)	0.211	0.709
Having a romantic partner	29 (59.18%)	12 (37.5%)	38 (52.78%)	32 (58.18%)	6 (35.29%)	0.156	**0.091**
Living in an urban area	45 (91.84%)	29 (90.63%)	67 (93.06%)	51 (92.73%)	16 (94.12%)	0.909	0.896
Number of rooms in the house	6.94 (2.70)	7.55 (3.49)	7.63 (2.86)	7.83 (2.76)	7.00 (3.16)	0.426	0.617
Religious beliefs	15.22 (7.95)	16.25 (7.57)	17.25 (9.11)	16.09 (8.51)	21.06 (10.24)	0.432	**0.099**
Neuroticism	31.55 (7.25)	33.59 (8.61)	36.81 (8.22)	37.65 (8.22)	34.06 (7.84)	**0.002**	**0.060**
Traumatic events	4.23 (2.59)	4.33 (2.32)	4.12 (2.32)	4.13 (2.13)	4.07 (3.01)	0.912	0.910
Stressful events before the onset of the study	17 (34.69%)	9 (28.13%)	37 (50.68%)	30 (53.57%)	7 (41.18%)	**0.054**	**0.067**
Stressful events during the study	0.17 (0.28)	0.27 (0.28)	0.19 (0.30)	0.20 (0.26)	0.09 (0.18)	0.334	0.118

*Model 1 refers to comparisons between men, HC users, and NC women, while model 2 refers to HC users, past users, and never users. For age, number of rooms in the house, religious beliefs, neuroticism, traumatic events, and having lived stressful events during the study (mean of the four time points), data represent group means (SD) in men, naturally cycling (NC) women, and women using hormonal contraceptives (HC). For ethnicity, education level, income, mental health diagnosis, physical health diagnosis, medication use, cohabitation with children, relationship status, living in an urban area, and having lived stressful events before the onset of the study, data represent group N (group %). For categorical variables with many subcategories (ethnicity, education level, income, cohabitation with children), data are shown according to the most frequent subcategory. Bold characters indicate covariates set at p < 0.100 that are included in the statistical analyses*.

### Measures

#### Hormonal Profile

In May 2020 (T0), participants declared their entire contraception history via self-reports, which allowed for their classification into one of four groups (HC users, never users, past users, men). For current and past HC users, the mean duration of their contraceptive use was 9.08 years (*SD* = 6.63; range of 0.5–28). Previous users had stopped using HC for a mean duration of 7.52 years (*SD* = 6.33; range of 0.5–23).

#### Psychological Distress

Psychological distress was measured using the Impact of Event Scale-Revised (38, 39) and the Depression Anxiety Stress Scales (40, 41).

##### Impact of Event Scale-Revised

The French version of the IES-R is a 22-item scale assessing perceived stress arising from a traumatic event (38). Items address PTSS felt in the last 7 days. The main question was adapted to assess COVID-related PTSS. The questionnaire is based on the English version developed by Weiss and Marmar (39). Items such as “Any reminder brought back feelings about it” (intrusion), “I stayed away from reminders of it” (avoidance), or “I felt irritable and angry” (hyperarousal) assess different PTSS that could arise in response to a traumatic event. Participants answered each item on a Likert scale ranging from 0 (not at all) to 4 (extremely). All answers were summed, yielding a total score between 0 and 88. Higher scores indicated more severe distress symptoms. This questionnaire has an excellent internal consistency, with a total score alpha value of 0.93 (38). The IES-R has been widely used to assess PTSS in the context of the COVID-19 pandemic [for a systematic review and meta-analysis, see (42)].

##### Depression Anxiety Stress Scales

The French version of the DASS-21 is a 21-item scale assessing the respondent's depression, anxiety, and stress symptoms in the last seven days (40). This version has been shortened and translated from the original 42-item English version developed by Lovibond and Lovibond (41, 43). Items include “I felt that I had nothing to look forward to”, “I felt scared without any good reason”, and “I experienced trembling (e.g., in the hands)”. Respondents could rate them on a scale from 0 (did not apply to me at all) to 3 (applied to me most of the time). Each item's score is doubled to allow for interpretation based on the original 42-item version. The DASS-21 includes three subscales (depression, anxiety, stress) and a total score, ranging from 0 to 126. The latter is obtained via the summation of scores from each of the three subscales. Similar to the IES-R, a higher score indicates more severe distress symptoms. The DASS-21 has shown to be reliable in adult populations (41, 44). The translated French version of the questionnaire exhibits acceptable internal consistency, with alpha values varying from 0.72 to 0.79 (40).

#### Questionnaire Completion

Participants answered the aforementioned questionnaires (among others) on Qualtrics, a secure online platform. A member of the research team sent participants a unique URL via email at each time point, where participants had 2 weeks to complete the questionnaires. During this period, participants could pause their questionnaire completion and continue later, with the condition that the questionnaires were completed within the 2-week time frame.

### Procedure and Timeline

**Figure 1:**
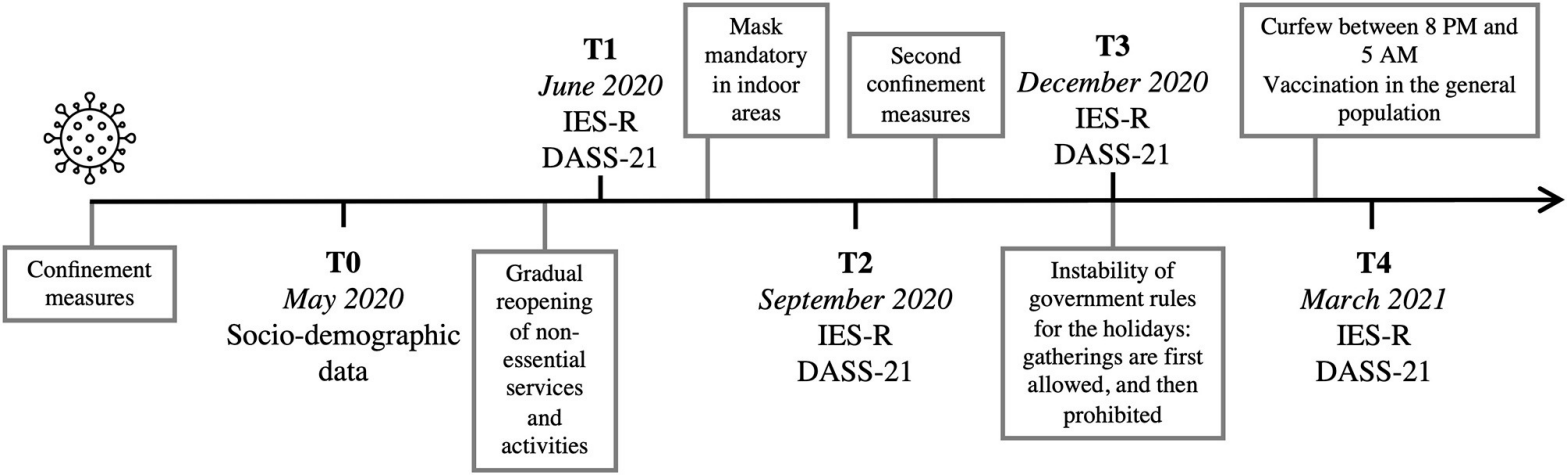
Timeline overview.

The first case of COVID-19 was reported in February 2020 in the province of Quebec, Canada. In March 2020, the Quebec government decreed several confinement measures and declared a public health emergency to limit the spread of COVID-19. We recontacted participants who had already taken part in one of our laboratory's studies. In May 2020 (T0), we obtained either verbal or written informed consent to pursue their implication in this added follow-up, in addition to the collection of socio-demographic data and potential confounding variables. Thereafter, participants were sent a series of questionnaire every 3 months in the following year: in June 2020 (T1), September 2020 (T2), December 2020 (T3), and March 2021 (T4; see Figure 1 for an overview of the study's timeline). Therefore, the IES-R and DASS-21 were completed at four different times (T1, T2, T3, T4). Participants received financial compensation proportional to their implication in this longitudinal study.

### Statistical Analyses

Data were examined where z-scores of ≥ ±3.29 were considered as outliers (45, 46). Using this criterion, <1.5% of the IES-R and DASS-21 scores were considered extreme. Participants whose scores exceeded DASS-21 clinical thresholds were recontacted and offered psychological resources. All extreme scores were winsorized with respect to study groups, using the next highest value of each group (47).

Prior to conducting the statistical analyses, we assessed the skewness and kurtosis of our main variables. Our data were normally distributed according to indices for acceptable limits of ± 2 and ± 7, respectively (48). Therefore, we used the raw values in our final analyses. All analyses were performed using SPSS 27 (IBM).

#### Preliminary Analyses

Covariates were selected from the analyses performed on sociodemographic variables. We tested variables that have been linked to psychological distress and on which our groups may have differed. Groups were compared with ANOVAs and chi-square tests for continuous and categorical variables, respectively. Variables that reached a *p*-value of < 0.10 were included as covariates in the analyses.

#### Main and Exploratory Analyses

To examine the impact of current HC use on psychological distress, our first subset of analyses compared HC users, NC women (never users, past users), and men, as these subgroups are generally compared when studying the role of hormonal contraception (49–56). We conducted linear mixed models to compare the evolution of PTSS (IES-R), general distress (DASS-21 total score), as well as depression, anxiety, and stress symptoms (DASS-21 scales) in HC users, NC women, and men. In the second subset of analyses, we explored long-term effects of HC use on women's psychological distress. To do so, we subdivided the NC women group using women's contraceptive history. As such, we obtained two subgroups: never and past users. This second set of linear mixed models was limited to women as we tracked the evolution of the same distress symptoms in HC users, past users, and never users. Restricted maximum likelihood (REML) was applied to allow for robust analysis with various and relatively small sample sizes (57). “Subjects” were considered as a random effect. Factors consisted of Time (four levels: T1, T2, T3, T4), Group (three levels in each subset—model 1: HC users, NC women, men; model 2: HC users, past users, never users), as well as the interaction term Time^*^Group. Between-subject and within-subject *post-hoc* comparisons were performed using Bonferroni's multiple comparisons test. Statistical significance was set at *p* < 0.05. An autoregressive covariance structure was first considered. Model residuals were inspected for normality and homoscedasticity by visual examination of residual plots. Deviations from homoscedasticity was observed for the main analyses. Therefore, a heterogenous version of the autoregressive covariance structure was selected. Visual inspection of residual plots for our exploratory analyses did not reveal any obvious deviations from homoscedasticity or normality.

## Results

### Main Analyses

#### Preliminary Analyses

Descriptive statistics for the three participant groups (i.e., women taking HC, NC women, and men) are shown in Table 2. Groups did not differ with respect to income, education level, relationship status, living in an urban area, number of rooms in the house, cohabitation with children, use of medication, having a diagnosis related to either physical or mental health, religious beliefs, traumatic events, and stressful events that occurred during the study (mean score of T1 to T4). However, groups were statistically different with regards to age (*p* < 0.001), having children (*p* = 0.030), and neuroticism (*p* = 0.002). Men were significantly older than both groups of women and had more children than HC users. As for neuroticism, NC women reported higher levels than men. Ethnicity was also quite unbalanced across groups (*p* = 0.084), as HC users were solely of Caucasian origin compared to NC women and men. Having experienced stressful events that occurred before the onset of the study was also marginally significant (*p* = 0.054). Pairwise z-tests did not yield any significant comparisons, although a higher proportion of NC women reported having lived a stressful event prior to the study compared to HC users. Given the non-randomized group assignment of this study and the potential interference with our main analyses, these five variables (age, having children, neuroticism, ethnicity, and stressful events prior to the pandemic) were considered as covariates in all subsequent analyses.

#### IES-R

**Figure 2:**
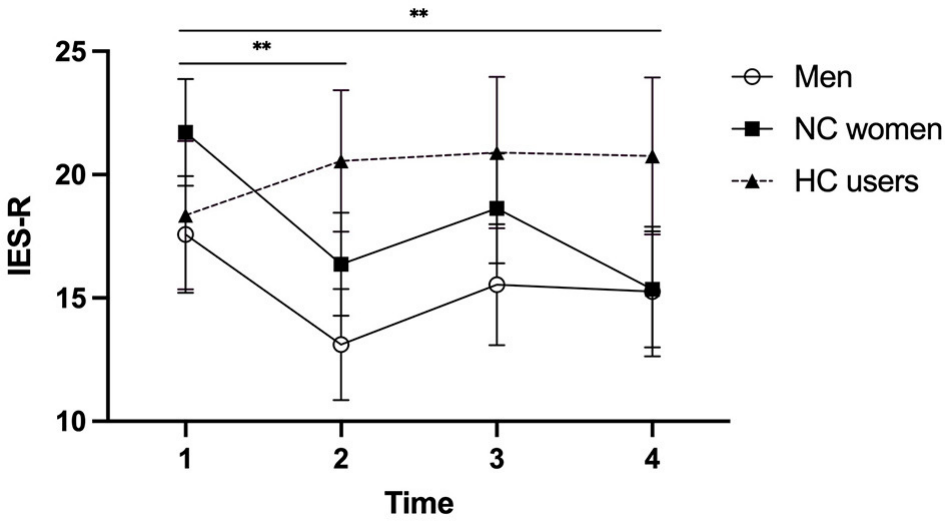
Post-traumatic stress symptoms (measured by the IES-R) of men, naturally cycling (NC) women, and women using hormonal contraceptives (HC) during the COVID-19 pandemic. A significant decrease from T1 to T2 was observed for NC women and men, as well as from T1 to T4 for NC women. Men, NC women, and HC users are illustrated by circles, squares, and triangles, respectively. Error bars represent standard error. **p < 0.01.

Comparing women taking HC, NC women, and men, our model for PTSS revealed a main effect of Time (*p* = 0.026) and a trend toward a Group effect (*p* = 0.079). A significant Time^*^Group interaction was found [*F*_(6, 236.03)_ = 2.212, *p* = 0.043], with women using HC reporting stable levels of PTSS across the four time points [*F*_(3, 41.36)_ = 0.486, *p* = 0.694] relative to NC women and men who showed a significant decrease from T1 to T2 (both *p*s < 0.01). NC women also exhibited a significant decrease between T1 and T4 (*p* = 0.009) (Figure 2).

#### DASS-21

**Figure 3:**
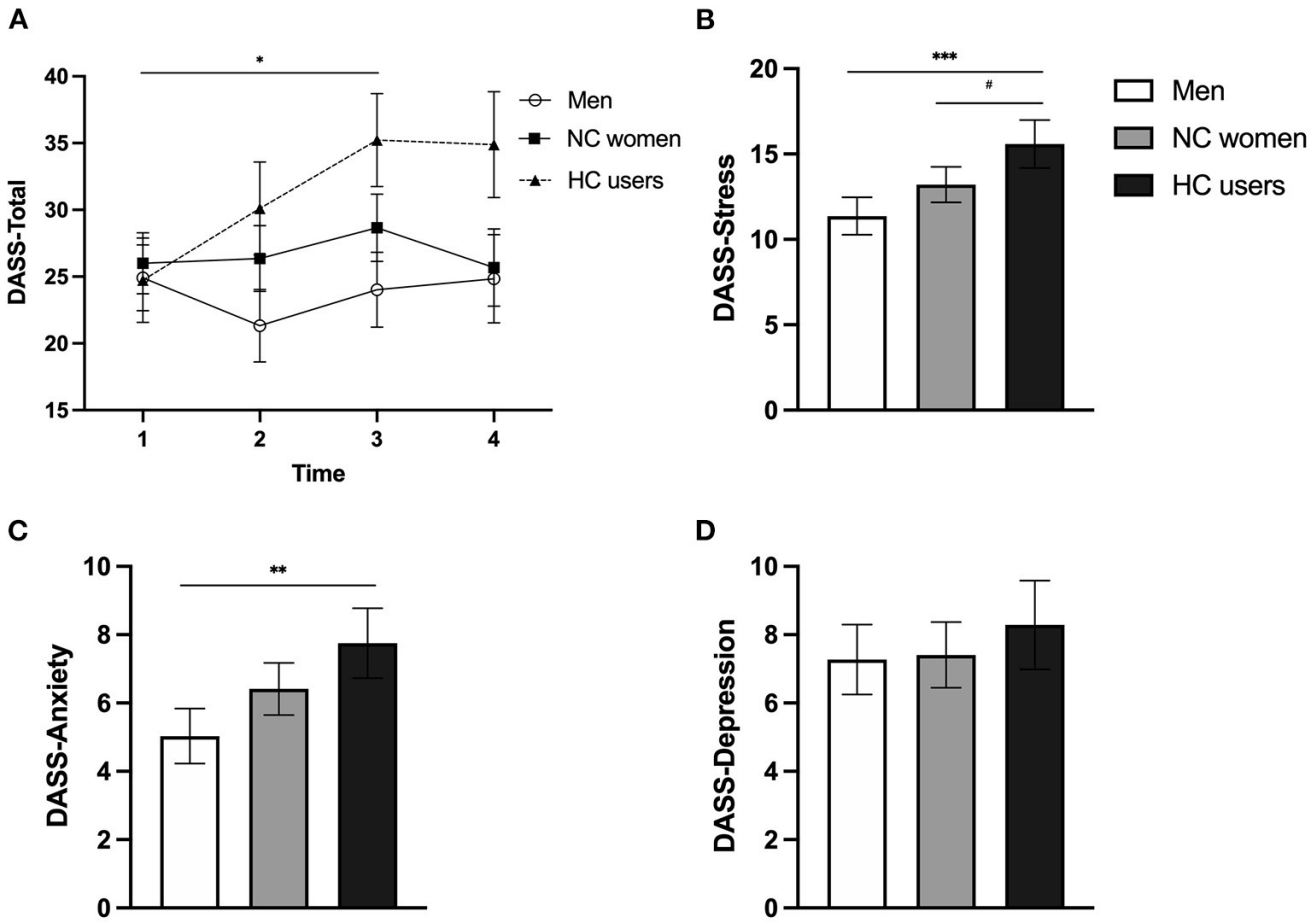
Symptoms of depression, anxiety, and stress (measured by the DASS-21 scores) of men, naturally cycling (NC) women, and women using hormonal contraceptives (HC) during the COVID-19 pandemic. **(A)** General distress evolution during the pandemic. Men, NC women, and HC users are illustrated by circles, squares, and triangles, respectively. **(B)** Mean stress symptoms. **(C)** Mean anxiety symptoms. **(D)** Mean depressive symptoms. Men, NC women, and HC users are illustrated by white, gray, and black bars, respectively. Error bars represent standard error. # < 0.08, *p < 0.05, **p < 0.01, ***p < 0.001.

As for the DASS total score (Figure 3A), results showed main effects of Time (*p* = 0.031) and Group (*p* = 0.013). The Time^*^Group interaction was significant [*F*_(6, 194.07)_ = 2.217, *p* = 0.043], with HC users reporting increasing levels of general psychological distress from T1 to T3 [*F*_(3, 38.74)_ = 3.428, *p* = 0.026] compared to NC women (*p* = 0.374) and men (*p* = 0.096).

When examining the three DASS scales, a main effect of Group was found for stress symptoms [*F*_(2, 165.62)_ = 6.816, *p* = 0.001] (Figure 3B) and for anxiety symptoms [*F*_(2, 153.80)_ = 5.483, *p* = 0.005] (Figure 3C). Both effects were driven by the fact that women using HC reported higher symptoms than men (*p* < 0.001 and *p* = 0.004 for the stress and anxiety scales, respectively). HC users also exhibited marginally higher stress symptoms than NC women (*p* = 0.076). The analysis also revealed a main effect of Time for stress symptoms [*F*_(3, 216.81)_ = 2.972, *p* = 0.033], where stress levels increased from T1 to T3 (*p* = 0.027). As for the depression scale, a main effect of Time was found [*F*_(3, 199.94)_ = 2.841, *p* = 0.039], indicating an increase of depressive symptoms from T2 to T3 (*p* = 0.052). No other main effect or interaction reached significance for the three subscales (*p*s > 0.125; Figure 3D showing the non-significant group comparison for the depression scale).

### Exploratory Analyses

#### Preliminary Analyses

As presented in Table 2, current HC users, past users, and never users were similar regarding income, education level, living in an urban area, number of rooms in the house, cohabitation with children, use of medication, having a diagnosis related to either physical or mental health, traumatic events, and stressful events during the study. Groups differed in terms of age (*p* = 0.010), with past users being older than never and current users. Groups also differed according to ethnicity (*p* < 0.001), with fewer never users being of Caucasian origin and more so of Asian, Arabic, and Hispanic origins than both past and current users. Having children was also a discriminant factor between the three groups of women (*p* = 0.009), with past users having more children than current and never users. Trends were found for neuroticism (*p* = 0.060) and having lived stressful events before the study (*p* = 0.067). Past users tended to report more neuroticism than current users. Although pairwise z-tests did not reach significance, more past users reported having lived a stressful event before the study than current and never users. Even though relationship status did not reach statistical significance (*p* = 0.091), a greater proportion of past users tended to have a romantic partner compared to current and never users. Finally, strength of religious faith also tend to vary between our groups (*p* = 0.099), with never users reporting higher religious beliefs than past and current users. To better evaluate the impact of HC on psychological distress in women, age, ethnicity, having children, neuroticism, stressful events before the study onset, relationship status, and religious beliefs were entered as covariates in the statistical models.

#### IES-R

With regards to the second set of analyses, results showed a nearly significant effect of Time for PTSS [*F*_(3, 258.45)_ = 2.612, *p* = 0.052] but no significant *post-hoc* comparisons emerged (*p*s > 0.400). No effect of Group (*p* = 0.623) nor Time^*^Group (*p* = 0.147) were found for PTSS.

#### DASS-21

**Figure 4:**
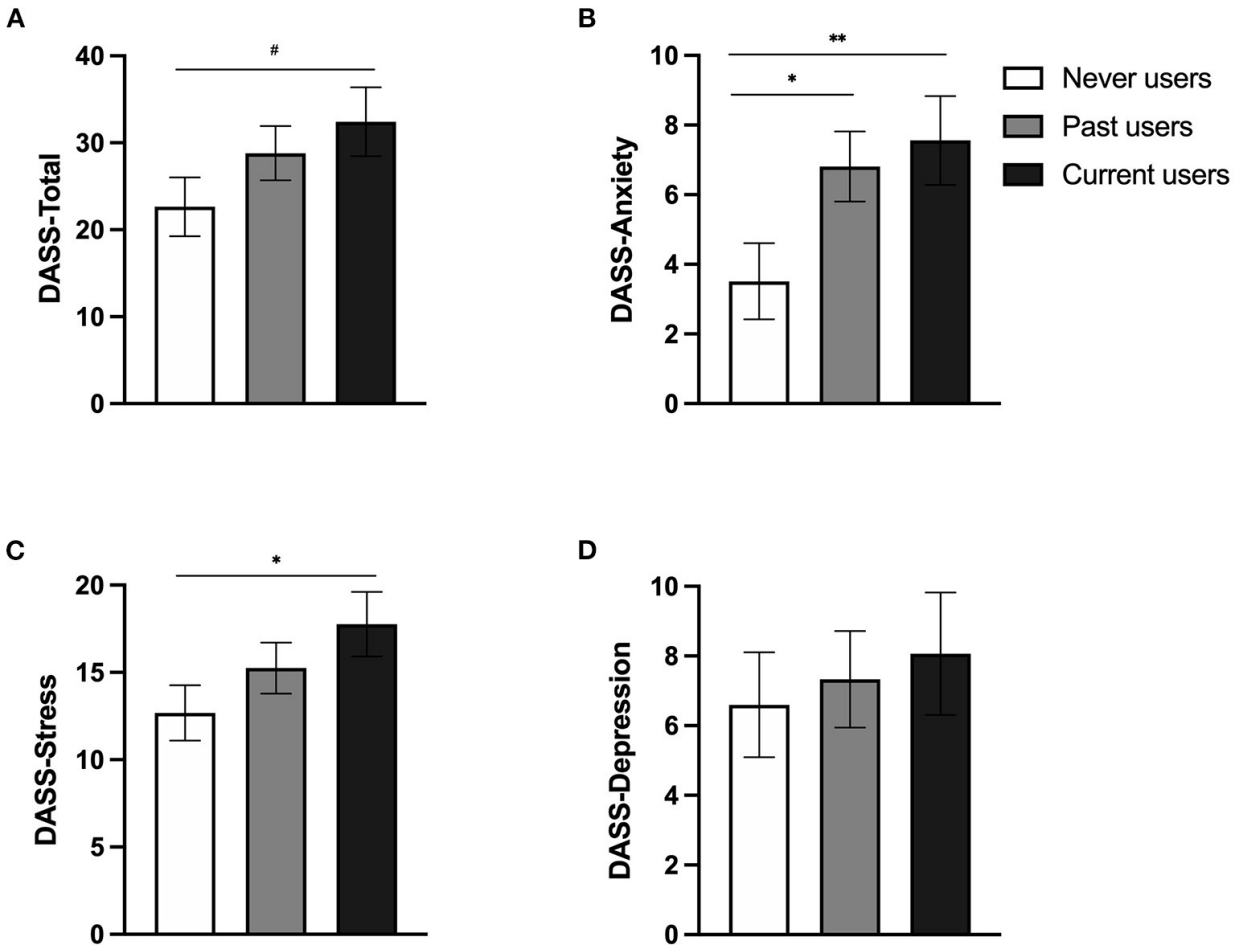
Symptoms of depression, anxiety, and stress (measured by the DASS-21) of current hormonal contraceptive (HC) users, past users, and never users during the COVID-19 pandemic. **(A)** Mean general distress. **(B)** Mean anxiety symptoms. **(C)** Mean stress symptoms. **(D)** Mean depressive symptoms. Never users, past users, and current users are illustrated by white, gray, and black bars, respectively. Error bars represent standard error. # < 0.08, *p < 0.05, **p < 0.01.

According to the DASS total score (Figure 4A), a main effect of Group was found [*F*_(2, 104.92)_ = 3.045, *p* = 0.052], with HC users experiencing more general distress than never users (*p* = 0.047). A trend toward an effect of Time was detected [*F*_(3, 261.02)_ = 2.516, *p* = 0.059] but no significant *post-hoc* comparisons emerged (*p*s > 0.084). No Time^*^Group interaction was found (*p* = 0.277).

A significant Group effect was revealed for the anxiety scale [*F*_(2, 107.96)_ = 5.242, *p* = 0.007] (Figure 4B). Irrespective of time, past and current HC users exhibited similar anxiety symptoms, which were significantly higher than those reported by women who never used HC (*p* = 0.006 and *p* = 0.015 when compared to HC and past users, respectively). The analysis for the stress scale also yielded a main effect of Group [*F*_(2, 104.22)_ = 4.054, *p* = 0.020], where HC users reported higher stress symptoms than never users (*p* = 0.022; Figure 4C). A marginal effect of Time was also found for the stress scale [*F*_(3, 259.18)_ = 2.351, *p* = 0.073] but no significant *post-hoc* comparisons emerged (*p*s > 0.121). No other main effect or interaction reached significance for the three subscales (*p*s > 0.095; Figure 4D showing the non-significant group comparison for the depression scale).

#### Parameters of HC Use

Past and current users were combined in order to explore the relationship between duration of HC use and psychological distress. Using hierarchical regressions, we entered covariates in the first step based on their correlates with each distress scale. Then, duration of use was entered in the second step. For each distress scale, no effect of duration of use was found (*p*s > 0.478). We also ran the same analyses according to the age of onset. For each distress scale, no effect of age of onset was found (*p*s > 0.417). Note that non-significant results are also obtained when running these analyses separately for past and current users.

## Discussion

In this study, we investigated the impact of hormonal contraceptives on psychological distress during the COVID-19 pandemic. Over a year, we followed distress evolution in women using HC, naturally cycling women (either never or past HC users), and men. Our models showed that HC intake was associated with elevated distress during the pandemic and that past HC users resembled current HC users in terms of anxiety symptoms. These findings suggest current and long-lasting effects of HC on psychological health.

In our first model, we compared men, NC women, and HC users based on the existing literature on hormonal contraception (49–56). First, we observed a lack of decreasing levels of PTSS in women using HC compared to NC women and men. As it has been reported that PTSS and COVID-related fear tends to decrease over time (7, 58), our result suggests that HC use is associated with a maladaptive response to a long-term stressor. This symptom maintenance could be explained by deficits in fear extinction, as HC use (i.e., oral contraception) was previously linked to poorer extinction recall, as assessed via laboratory experiments (28) and exposure-based treatment (29, 59). We also found an increase in general distress over time in women using HC compared to NC women and men. Finally, HC users were more stressed and anxious than men overall. The finding that HC users stood out by showing higher distress patterns supports our hypothesis that psychological impairment is associated with current HC intake. Distress differences between men and women relative to the COVID-19 pandemic were reported extensively in previous studies (5, 14, 15, 60–63). Interestingly, our data explains this gap by extending beyond psychosocial factors related to feminine gender (e.g., job loss, virus exposure, domestic responsibilities) (17). Indeed, it emphasizes the importance of also considering biological sex-related factors in order to understand what may render women more vulnerable to psychological distress and mental health disorders. In addition, contrary to what is generally assumed by most studies, our findings suggest that women might not represent a homogenous group. When hormonal status is considered, the lack of differences between NC women and men is noteworthy. As no other COVID-19 study to our knowledge has considered the role of sex hormones, this raises the possibility that previous results regarding psychological distress in women during the COVID-19 pandemic might be driven or accentuated by samples containing women using HC.

Accordingly, current HC use could exert its negative effects on mental health via different neurobiological mechanisms. First, fear and other emotions potentially evoked by the virus outbreak, are regulated through limbic circuitry linking the amygdala and prefrontal cortex, among other structures. Dysregulation of this circuitry has often been associated with mood, anxiety, and fear-related disorders (64). Animal studies have shown that the amygdala and prefrontal cortex have a particularly high density of sex hormones receptors. In humans, HC studies focused mostly on oral contraceptives and showed that its use could be linked to emotional regulation (65, 66). Graham and Milad (2013) found that HC use was negatively associated with fear regulation in women and female rats. In ovariectomized rodents, estrogen drops were associated with a decrease of dendritic density in the prefrontal cortex (28). Another study found a reduction in the volume of the left amygdala and gray matter following a 3-month use of oral contraceptives. Connectivity between the amygdala and prefrontal cortex was also shown to be altered (67). Volume of many other brain structures playing a role in emotion regulation seems to be altered following HC intake (e.g., insula, anterior cingulate cortex, orbitofrontal cortex) (66). Also, although exact mechanisms remain unclear, endogenous female sex hormones seem to play a role in anxiety reactions. Among other things, allopregnanolone (a progesterone metabolite) acts on GABA receptors, leading to either anxiogenic or anxiolytic effects, depending on metabolite and receptor concentrations (65, 68). As synthetic doses of progesterone lead to chronically low concentrations of endogenous progesterone, HC use may result in alterations of anxiety regulation (65). Finally, it is worth noting that sex hormones interact with the HPA axis and may modulate stress regulation (65, 69). Indeed, oral contraceptive users and non-users exhibited different neural responses following cortisol (stress hormone released by the HPA axis) administration (52). Interestingly, fMRI data suggested that, in the occurrence of a stressful situation (in this case, provoked by cortisol administration), oral contraceptive users had enhanced hippocampal activity during fear learning compared to their naturally cycling counterparts and men (52). Another study indicated that oral contraceptive users show blunted salivary cortisol reactivity in response to a psychosocial stress relative to NC women in the luteal phase and men (70). Thus, the stress response of HC users and non-users seem to differ according to biomarkers such as cortisol (70). These interactions between sex hormones and the HPA axis could be linked to differential distress symptomatology among HC users and non-users during the COVID-19 pandemic. In sum, the results of this study align with the existing work on mechanisms underlying the association between HC use and mental health. Due to the longitudinal design of this study, our results also support the idea that chronic use of HC (rather than its synthetic compounds) may have the most influence on psychological distress.

Regarding other results from our main analyses, no sex differences were found in terms of depressive symptoms in our sample. Women (HC users or NC women) did not show more depressive symptoms than men during the COVID-19 pandemic. This is an intriguing result as women generally report higher levels of depression than men (10, 60). However, another study conducted during the COVID-19 pandemic also found equivalent depressive symptoms between men and women (71). Pre-pandemic analyses were carried out on this study's sample to assess differences between men and women on depressive symptoms. In controlling for the time elapsed between the completion of the initial questionnaire and T1 of this COVID-19 study, Arcand et al. (in preparation) showed that women scored higher than men on depressive symptoms before the pandemic. Among the numerous ways to explain this finding, it is possible that women in our sample tended to mitigate their depressive symptom levels (e.g., less fatigue, more time to do meaningful activities) compared to men. Another possibility is that men may have had a larger increase in depressive symptom levels in response to the pandemic (e.g., difficulty reaching out to others, social isolation) compared to women.

When examining the main effects of time in our first subset of analyses, the context around T3 (December 2020) seemed to have particularly deleterious effects on mental health. Indeed, stress symptoms increased from T1 to T3, depressive symptoms increased from T2 to T3, and HC users showed greater general psychological distress levels at T3 compared to T1. Apart from these statistically significant comparisons, all psychological distress measures were heightened at T3. As illustrated in Figure 1, the Quebec government first announced that small gatherings would be allowed during the holidays but retracted this decision soon thereafter. Measurements from T3 coincided with these contradictory announcements. Thus, the changing governmental measures, declaration of a prolonged confinement period (immediately following an increase in hope that we would have an “almost normal” holiday period), and the beginning of winter (72) may have contributed to the decrease in psychological wellbeing.

Despite the relevance of studying the impact of current HC use, investigating its long-lasting effects is of great interest to better understand how sex hormone modulation can affect the brain and behavior of women. As a small body of evidence supports this standpoint (33, 35–37, 73), it appeared essential to explore if previous use of HC could induce a durable influence on psychological health. Thus, according to our secondary objective, our results showed that current and past users exhibited higher anxiety than never users, a pattern that was also found for stress symptoms with current users reporting more stress than never users, and past users not differing from any of the two groups. These findings partially support our hypothesis. In fact, our results suggest that a durable trace of HC could be suspected, particularly for anxiety manifestations, in response to a chronic stressor such as the COVID-19 pandemic. Two potential pathways have been highlighted according to previous studies on HC. First, prolonged intake of HC, therefore abolishing high levels of sex hormones for a considerable amount of time, could underlie a potential mechanism on how HC impacts brain anatomy and function. Indeed, Pletzer et al. (37) have linked the duration of previous oral contraceptive use to gray matter volumes of subcortical structures. Although women were no longer using any HC, longer durations of oral contraceptive use were associated with larger hippocampi and basal ganglia (37). Second, timing of HC intake could also be responsible for long-lasting changes in the brain. Recent investigations support this idea, where pubertal onset of oral contraceptives was associated with differences in stress reactivity, brain structures, and functional connectivity compared to an adult onset of use (73, 74). In knowing that adolescence represents a crucial time frame for brain development and reorganization (75, 76), it is plausible to consider a disruption of these processes by HC intake (e.g., synaptic pruning, myelination, dendritic elaboration). However, duration of use and age of onset were not associated with psychological distress in our study. This contradicting finding could reflect the complexity of obtaining reliable data on these retrospective data. Indeed, women often switch from one HC to another, pause their HC for various amount of time, and might also have difficulty recalling with precision their history of use (37). Of note, data about history of use were all collected online, which could have prevented to obtain the level of details needed to accurately reflect the total duration of HC use. For example, some women with a complex history of HC use might not electronically report their duration of use as accurately as if they spoke on the phone with a research assistant trained to guide them into taking time to think, summarize what has been said, and revalidate the information.

Yet, if there is truly no impact of these two HC parameters, it is plausible that other factors such as interrupted use of HC could hamper the relationship between duration or age of onset and psychological outcomes.

For most of our scales, no clear distinction was observed between women who previously used HC and those who never did. Reversibility of the effects of HC is undoubtedly a natural phenomenon that occurs as a consequence of brain plasticity and homeostasis (37, 77–79). As such, it is essential to remain careful when making assumptions about the long-term potential of HC. As mentioned, women frequently pause their HC. It would be interesting to develop a more comprehensive approach by taking into account the total duration of use with respect to the time spent without using an HC. It would also be of great relevance for future investigations conducted in former HC users to consider the amount of time elapsed since HC discontinuation. This would help to get a better grasp of the dynamic interplay between onset and duration of HC use and its cessation duration. Longitudinal studies carried out over extended periods of time would also allow for an improved comprehension of the balance between both the durable and reversible effects of HC.

The present study has limitations. First, we used data from a larger study where individuals were recruited based on their past participation in a study at our laboratory. Consequently, our final sample was rather small and group formation was made *a posteriori*, as it was constrained by the context of the pandemic. Nonetheless, linear mixed models can manage unbalanced designs in longitudinal datasets, therefore allowing for a more robust examination of unequal sample sizes and prevention of a decrease in power due to attrition (80, 81). Second, given that our sample was not selected for the purposes of the current study and that group assignment was non-randomized, it cannot be assumed that there was equivalence across our comparison groups. Despite controlling for several covariates, other non-controlled factors could bring unwanted variability to the results such as heterogenous hormonal events. For example, experiencing one or many pregnancies, taking hormone-related medication, or having used or currently using different HC methods could decrease internal validity and statistical power. Even if combined oral contraceptives were the most frequently used method in our sample, it is still unknown if all HC methods provide the same effects. As our study did not allow for this level of precision, this highlights the importance of refining future methodologies in order to unveil specific mechanisms. Studying HC onset use and mental health prospectively would also provide stronger evidence of the deleterious impact of HC. Additionally, distress scores observed in this study were below the clinical threshold of the scales. Thus, the negative impact of HC may not impair the normal functioning of women, suggesting that clinical significance is weak. Nevertheless, it remains quite informative to see that HC could affect women in the general population to some extent and that the effects of HC seem to persist after cessation of use.

In terms of its strengths and contributions, our study highlights the value of adding biological factors to further understand gender differences in mental health. It also covered a considerable time frame, which allowed for a more complete assessment of distress evolution during a chronic stressor such as the COVID-19 pandemic. We have taken into account numerous aspects at the bio-psycho-social levels with the intention of isolating the impact of HC on psychological distress. Moreover, this is one of the few studies that has explored the long-lasting effects of HC by investigated both never and past HC users. Our results converge with the limited literature on the topic and promote avenues for further research in the field. Considering not only current use of HC but also its history could provide insight for understanding why certain subgroups of women are prone to higher psychological distress than men.

## Conclusion

The COVID-19 pandemic challenged everyone's mental health, yet some people have adapted to this situation better than others. Thus, we sought to examine whether certain subgroups of the population, depending on their HC use, were more vulnerable than others in a context of chronic stress. Our results support our hypotheses as HC use seems to have worsened distress symptoms among our participants. Moreover, it is worth noting that HC use may have a prolonged impact on mental health, even years after women ceased their intake. These results highlight the importance of conducting further research that considers not only psychosocial, but also biological factors like sex hormones as potential determinants of mental health outcomes.

## Data Availability Statement

The raw data supporting the conclusions of this article will be made available by the authors, without undue reservation.

## Ethics Statement

The studies involving human participants were reviewed and approved by Centre intégré universitaire de santé et de services sociaux de l'Est-de-l'Île-de-Montréal. The patients/participants provided their written informed consent to participate in this study.

## Author Contributions

AB and MFM contributed to the conception and design of the study. AB performed the statistical analyses. AB, LMD, and JF wrote sections of the manuscript. All authors contributed to manuscript revision.

## Funding

This project was financially supported by the Fonds de recherche du Québec - Santé (FRQS; #251601, #265447), the Canadian Institutes of Health Research (CIHR; #FRN169080), and the Natural Sciences and Engineering Research Council of Canada (NSERC; #RGPIN-2018-06082). MFM holds a salary award from the FRQS. AB holds a M.Sc. Scholarship from the FRQS. LMD holds a M.Sc. Scholarship from the CIHR. JF hold a Ph.D. Scholarship from the FRQS.

## Conflict of Interest

The authors declare that the research was conducted in the absence of any commercial or financial relationships that could be construed as a potential conflict of interest.

## Publisher's Note

All claims expressed in this article are solely those of the authors and do not necessarily represent those of their affiliated organizations, or those of the publisher, the editors and the reviewers. Any product that may be evaluated in this article, or claim that may be made by its manufacturer, is not guaranteed or endorsed by the publisher.
